# The rules of actionable reporting

**DOI:** 10.1186/s13244-026-02429-8

**Published:** 2026-10-25

**Authors:** Paul McCoubrie

**Affiliations:** Radiology Department, Southmead Hospital, Bristol, United Kingdom

**Keywords:** Actionable reporting, Rules, Communication

## Abstract

**Abstract:**

Actionable reporting in radiology is a concept that has gained traction since its inception in 2014. The notion is simple: radiology reports should easily translate into clinical action, particularly when the study is abnormal. This is not so simple. But the process of producing an actionable report is as crucial as the report itself. The process should result in a radiology report that consistently results in information being provided to the referrer that results in improved patient care. This review article presents an evidence-based critique of the concept, demonstrates the numerous pitfalls, illustrates points of gold-standard practice (illustrated as ‘Rules’) and presents a coherent model of the overall process of actionable reporting, which flanks the crucial central element of an actionable report.

**Key Points:**

**Question** The actionable reporting process is highly dependent on cooperation and coordination of many healthcare staff, IT systems, and different departments, with the radiologist at the heart of it; it should be seen as a multistep process where each step is crucial and interdependent with the overall goal of better patient outcomes via better systems of work.

**Findings** The skilful production of an actionable report is central to this process and remains the responsibility of the individual radiologist.

**Critical relevance statement** Fundamentally, actionable reporting represents the current best practice in radiology reporting. It should be the goal of every radiologist to ensure their reports are actionable and their reporting workflow matches that of actionable reporting.

**Graphical Abstract:**

**Figure:**
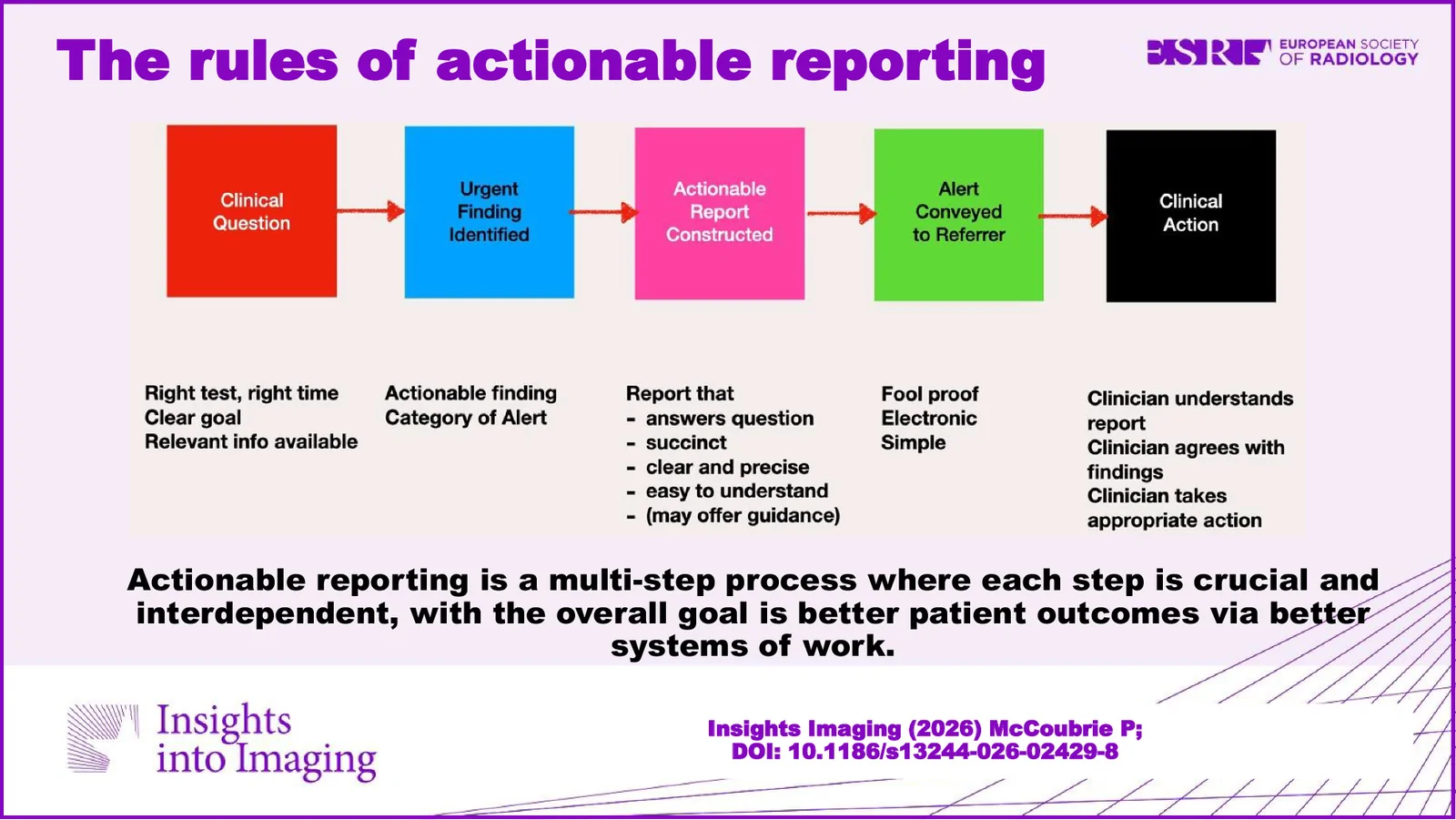

## Introduction

‘Actionable reporting’ is a phrase in common use in radiology but is a relatively modern concept, first coined in 2014 by the American College of Radiology’s (ACR) Actionable Reporting Work Group [1–3]. However, many definitions of the term exist, and full adoption of the model remains limited outside the USA. Perhaps, unsurprisingly, there is criticism of the ACR model.

This paper will critically examine the concept, detail its evolution, summarise the evidence behind its contemporary use, as well as detailing pitfalls and unintended consequences. Best-evidence recommendations for practising radiologists will be presented, phrased as ‘Rules’; guidance written as easy-to-remember idioms, a method that has proven popular [4, 5].

### Modern duties of a radiologist

One of the key skills of a radiologist is to communicate the results of radiological investigations to the referring clinician [6]. To communicate poorly is to fail our colleagues and, in turn, the patient. Radiologists don’t always get it right; key information is not always articulated clearly and accurately. In addition, the report communication systems of work must be foolproof too. Perfectly-crafted reports are of little use if the reports are not delivered to the referrer promptly and reliably [7].

Radiology reports should also facilitate the next steps necessary after the test. The World Health Organisation has flagged a global problem with suboptimal test follow-up [8], resulting in serious lapses in patient care. Managing the follow-up of test results is complex. There are multiple steps, multiple individuals, and multiple information systems, creating an environment that increases the risk of error [9]. Radiologists have a role in reducing poor adherence to follow-up, particularly with critical test results, making explicit recommendations in their report so that subsequent management steps are clear to the referrer.

There is a multistep process between report construction and referrer taking appropriate action based on the report, with the possibility of failure at each step, with resultant patient harm as a result. It is perhaps not surprising that failure of communication is one of the more common causes of litigation in radiology [10–13]. Of note, most claims resulting from failure to emphasise the urgency or unexpected nature of an abnormality [14].

This latter notion is central to the concept of actionable reporting. Ensuring that referrers receive, understand, and act on abnormal reports was always vital, but there has been a subtle change in modern radiological practice. The duty of a radiologist used to end at the sign-off of the radiology report. If appropriate action didn’t occur after the report was issued, this was the sole fault of the referrer. Whilst referrers still have a duty to read the reports of scans that they have requested, radiologists now have the additional medicolegal duty of ensuring that urgent reports are definitely received by the referrer [15, 16].

Whilst the referrer has always been and still is the primary target for the radiology report, the patient themselves may have direct access to their radiology reports and are therefore important secondary consumers [17]. There are many methods of making radiology reports more patient-friendly, most of which involve additional action by radiologists, difficult in a resource-constrained healthcare system. Two of the key messages from patient feedback are that they find radiology reports difficult to comprehend as they contained “unclear or technical language” and were “too long” [18], reinforcing the need for clear and succinct reports.

Accordingly, modern radiologists need to think of several issues prior to final report sign-off:

1. Should the report be communicated over and above normal channels of communication?
2. And if so, what is the urgency of this communication and what method of additional communication should be used?
3. If the report is to be communicated urgently, who is the most appropriate clinician to deliver it to? (NB: This may be different to the original referrer and may need to go to multiple individuals, even a group or team.)
4. Any additional communication should be documented in the report (e.g., date, time, method and individuals contacted)
5. Any scan review or subsequent clinico-radiological discussion that leads to a different opinion of the radiological study should be documented as an addendum, including who the scan was discussed with and when
6. The report is likely to be read by the patient at some stage—is it concise, clear and free of unnecessary jargon?

It is these concepts that lie behind the rise of actionable reporting.

### ACR recommendations

The ACR’s 3 key papers [1–3] lay out a specific model of what actionable reporting should look like, summarising, “The path to actionable information starts at the beginning of the imaging value chain by choosing the right test for the right patient at the right time and ends when a succinct structured report … is delivered to the requesting caregiver”. These papers then detail the key components of an actionable report.

- Clear clinical question: This is crucial for two reasons. First, it ensures that the right study with the right protocol is performed, giving the radiologist the best chance of a diagnostic study. Second, the report can be optimised to address the clinical question, “…making the report more meaningful, specific and thus actionable”.
- Accessible clinical data: All prior radiological studies, biomarkers (e.g., blood work) and clinical data (e.g., surgical history) should be easily accessible to the interpreting radiologist, especially in the light of frequently inadequate data on radiology requests.
- Succinct, structured reports: Reports should be easy to read, and key information should be “positioned in the expected locations”. A standardised lexicon can improve clarity of communication by reducing inter-radiologist report variability.
- Recommendations: Any additional tests or other recommendations should be as evidence-based as possible or congruent with published guidelines.
- Timely communication of critical results: Abnormal findings that need additional communication are divided by urgency into those that should be communicated within Minutes (Category 1), Hours (Category 2) or Days (Category 3). Extensive examples of Category 1, 2 and 3 abnormalities are listed.
- IT support: The ACR stressed the importance of reliable IT systems to support the delivery of Category 1–3 reports. Systems should be easy to use and fit in with radiologists’ workflow. Report tracking and audit trails are vital.

### Recommendations from the United Kingdom

The Second Edition of the Royal College of Radiologists (RCR) ‘Standards for Interpretation and Reporting of Imaging Investigations’ was released in 2018 [19]. These standards embraced the concept of actionable reporting but differed from the original ACR model. Their definition of actionable reporting was much simpler, stating, “The report should answer the clinical question. When an abnormality is described, a tentative or differential diagnosis should be provided”. The RCR was unflinching in mandating that 100% of reports should answer the clinical question and, where an abnormality was described, 100% of reports should provide a likely diagnosis. The RCR made no specific recommendations on the classification of abnormalities nor any mandates on additional communication (other than to “comply with local mechanisms to alert referrers”). This guidance covered all reports, not necessarily just reports that contained abnormalities.

Duncan et al [20] documented widespread variation in UK practice in radiology communication systems, noting numerous reports of patient harm that had resulted from failed communication. The Health Services Safety Investigations Body subsequently led a national safety investigation and, in 2019, recommended a cross-speciality working party to develop recommendations to reduce risk from communication failure [7].

Subsequently, the Academy of Medical Royal Colleges (AoMRC) released “Recommendations: Alerts and notification of imaging reports” in 2022. This document was a cross-profession solution to the problem of radiology reports not being received, understood and prompting appropriate action [21]. It is a pragmatic document that sets out how healthcare organisations should handle urgent radiology reports. It specifically contained three definitions of urgent reports: (1) unexpected cancer, (2) critical findings and (3) significant addenda. Notably, it flags up how reporting communication systems are an issue for healthcare organisations rather than purely a problem for individual radiologists. However, the sensible recommendation of forming a hospital or organisation-wide ‘results coordination team’ seems to have largely been ignored—I cannot find a single organisation that has developed such a team.

### Emphasis on an actionable conclusion

Radiologists acknowledge the importance of including a well-formulated summary in complex radiology reports. Specifically, the conclusion or impression shouldn’t simply restate the body of the report but should be a thoughtful synthesis of the meaning of the findings [22]. Short reports less than four lines long may not need a conclusion, but it is the only section that busy clinicians are guaranteed to read, particularly if the report is long [23]. More recent authors have emphasised that whilst the body of the report may contain non-actionable descriptions or technical detail, the conclusion itself should be actionable by providing appropriate recommendations [24, 25].

### A modern definition of actionable reporting

**Fig. 1:**
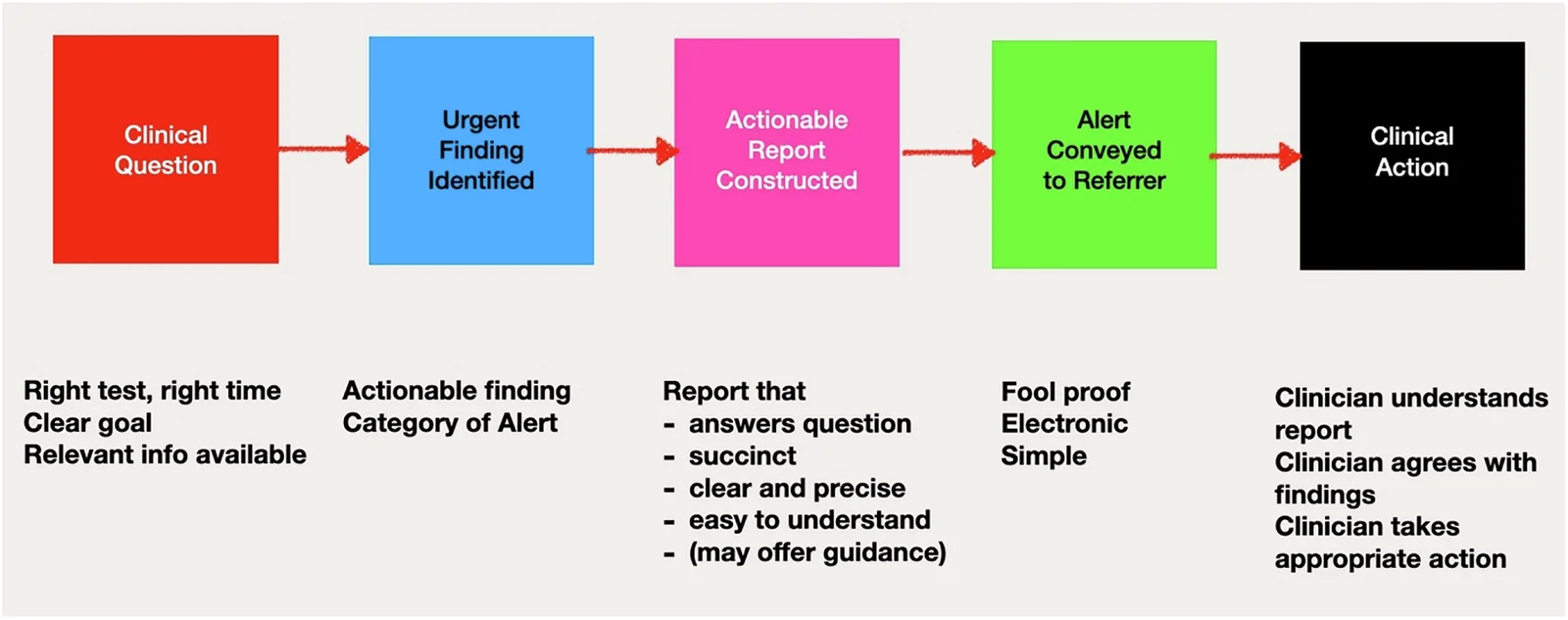
The multiple consecutive components of actionable reporting

Although the literature is sparse and definitions of terms are variable, a consensus does emerge. Actionable reporting should be thought of as a linear multistep process (Fig. 1). The discovery of an urgent finding and the construction of an actionable report are just subsections of the whole actionable reporting process.

From this, a working definition of actionable reporting can be stated as:

“Actionable reporting starts with a request for an appropriate investigation for the right patient, which is then performed at the right time and finishes when a clear, succinct, readable report containing appropriate guidance is safely delivered with appropriate urgency to the requesting clinician”.

## The 5 steps to producing an actionable report

### Step 1—Deciphering the clinical question

The modern radiologist is best seen not as a ‘gatekeeper’ but more as a ‘steward’, solving problems collaboratively [26]. By working closely with others, radiologists can ensure that each patient gets the right test at the right time to help advance their care. Radiologists are unique in that they understand both the clinical context and how radiological testing can help each patient. In reality, this means evaluating radiology requests to ensure they are clinically appropriate and protocoling each one to ensure the correct scan modality and scan protocol are selected.

Do radiologists need to scrutinise and protocol every investigation? Obviously not: it would be unfeasible. Other trained staff can do this, particularly if aided by clear flowcharts and written guidance. Radiologists may be best reserved for expensive, complex or invasive tests. But it is clear that radiologists should not step away from screening and protocol requests if they care about actionable reports.

It is established that the provision of a good clinical history and clearly articulated questions improves the accuracy and confidence of radiology reports [27]. The review by Castillo et al highlights that this is more likely to result in a more focused and more relevant radiology report. It also makes reporting quicker as the radiologist doesn’t have to spend time trying to deduce the question or look for details in priors, pathology results or other resources. The increased clarity of the clinical circumstances also allows the radiologist to reduce or even eliminate hedging and vagueness, which, as Berlin clearly articulates, has been a problem for over a century [28].

Occasionally, radiology requests arrive without a clinical question and either scant or irrelevant clinical details. How dogmatic should radiologists and other radiographic staff be about accepting such requests? Should they return every inadequate request? Or only for expensive, complex, or invasive tests? It is a judgement call—radiologists don’t want to be obstructive or unhelpful, but at the same time they should insist on basic professional standards so as to produce an actionable report. I would argue that blank or inadequate requests should be weeded out before the test is performed by empowering bookings and radiographic staff to challenge such requests, asking for further clinical details from either the referrer or the patient. Radiologists need to support such measures, as without them, they have little hope of producing an actionable report.

### Step 2—The problems of alert categorisation

Whilst all authors agree that unexpected cancers and critical findings should be communicated urgently, there is controversy about how this works in practice. See et al [29] adopt a pragmatic tone, acknowledging that the boundaries between ‘critical’, ‘urgent’ and ‘unexpected significant’ findings are not clearly defined and somewhat controversial. They note the inherent subjectivity of the categories, which vary by clinical context, location and radiological finding. One of their most valid points, in my opinion, is that radiological findings are often not definitive and rarely pathognomic. The messy and ill-defined reality of clinical practice makes a binary choice of ‘alert: no alert’ often difficult to use.

Visser et al [30] similarly found ambiguity in the ACR categorisation system, with only a moderate inter-rater agreement (kappa = 0.7), with a 19% disagreement in categorisation. They also found a high overall prevalence (33%) of ACR category 1–3 actionable findings: fully 46% of emergency reports were actionable. Hence, if ACR advice were to be fully implemented, nearly half of emergency radiology reports would be sent through with an alert.

This specific finding has led to criticism. Waite et al [16] noted that referrers are bombarded with non-urgent clinical alerts, leading to ‘alert fatigue’ with the risk that referrers may inadvertently ignore genuine urgent alerts. Furthermore, they criticise the use of alerts for non-acute findings, stating that “the primary motivation behind alerts … is to avoid litigation rather than promote patient safety”.

I call this ‘The Radiologist Who Cried Wolf’ problem, after the classic Aesop Fable of the shepherd boy who tricks villagers into believing a wolf is attacking, only for them to ignore his real pleas later. If an alerting system is overly inclusive, alerts lose their effectiveness through over-saturation, and the system fails.

There are three further criticisms of alerting systems which were noted in the European Society of Radiology (ESR) guidance in 2012 [31]:

1. Unread reports: Alert systems encourage referrers to read only reports that are flagged. Unflagged reports are demoted as unimportant and can go unread. Which means that half of all reports can go unread. It might be argued that this doesn’t matter, as a report that contains no actionable findings doesn’t need to be read. However, there are many circumstances where a normal or near-normal report needs to be read. The most common are (i) normal findings, but the report contains important guidance; (ii) minor or equivocal findings that need follow-up (but are not abnormal enough to generate an alert) and (iii) normal results, but the test is insensitive for a specific finding and further investigation is warranted.
2. Radiologists liable for everything: When an alert system is in place, the radiologist is blamed if they fail to flag up abnormalities. Which can be an appropriate chastisement. However, in my experience of cases of potential medical negligence, radiologists are frequently blamed, irrespective of what actually happened. Radiologists are frequently alleged to have not alerted the referrer rapidly enough, not alerted by the correct route, not alerting the right person and so forth. Such allegations are strong drivers of defensive behaviour. A defensive radiologist flags each and every abnormality, alerting multiple individuals, triggering urgent pathways, advising further investigation and specialist discussion, just in case.
3. Productivity costs: Every alert takes additional time and resources, even if delegated to clerical staff. If alerting systems are not easy to use, the loss of productivity is something that resource-constrained systems can ill afford.

This means that any alerting system needs to be robust and effective but should be used sparingly, reserved for serious or unexpected findings, particularly where action needs to be taken rapidly. The pragmatic advice of the UK AoMRC 2022 report is a good example of this [21].

### Step 3—Construction of an actionable report

There are many papers on the best way to construct radiology reports [6, 15, 22, 24, 32–36]. The ACR papers map out the essentials of an actionable report [1–3], and the latest RCR guidelines are sensible too [25]. However, the radiologist wanting to make their reports actionable may struggle for explicit advice. For this reason, I have constructed evidence-based guidance in the form of Rules (see Box 1).

Reporting is a learnable skill but often isn’t taught explicitly. The ability to construct an actionable report doesn’t always correlate with interpretational ability either: some of the best radiologists produce some of the worst reports. But radiologists of any age can improve if they open their minds to new ideas. Even the oldest radiologists can unlearn bad habits and make their reports more actionable. All radiology reports can be made actionable if they obey the following rules.

#### Rule 1—Answer the clinical question

The first step is to understand the question being asked. Sometimes this is explicitly stated on the written request, sometimes the radiologist has to deduce the question from the information supplied, and sometimes it isn’t clear at all. When met with missing information, unintelligible acronyms or obtuse clinical details, the radiologist has to look at prior studies, pathology results and other records.

Once the clinical question has been identified, the radiologist should ensure they answer it to the best of their ability. An important subtext in answering the question explicitly is to reassure the referrer (and, in turn, the patient). Bosmans et al [33] showed that half of the referrers believed that if a particular feature was not explicitly mentioned, then the radiologists might not have looked at it. Therefore, answering the clinical question is to issue a report that referrers are more likely to trust. Hence, answering the clinical question is a basic professional duty of a radiologist.

#### Rule 2—Conciseness is king

There is no ideal length of a radiology report. It varies by study complexity, radiologist experience, confidence, personal style and cultural norms. As Michael Rothman observed, “…the length of the body of the report depended on the number of findings, whereas the length of the conclusion varied with my ability to make sense of the findings” [37]. However, Leo Martin wrote in 1982, “A report need not always be brief but it can always be concise” [38]. This is still true over 40 years later. Even in the face of uncertainty and complexity, there is a wide consensus that radiologists should endeavour to express themselves as concisely as possible [1, 6, 15, 22, 24, 25, 39, 40].

There are several reasons for advocating conciseness in radiology reports. First, busy referrers are encouraged to read the report and not just skip to the conclusion. Second, conciseness instils confidence and engagement in the reader. There is evidence that referrers view long and complex reports as less reliable, possibly because they feel the radiologist may be obfuscating [41]. Third, conciseness is a cornerstone of good reporting. Without it, all further efforts are in vain. A wordy and rambling report is virtually always unreadable, lacking in clarity and is rarely actionable.

Bosmans et al highlighted a paradox about brevity in reports, pointing out that although concise reports are favoured by referrers and radiologists alike, extremely brief reports such as ‘No abnormal findings’ are not favoured, particularly with complex examinations [33]. Hence, reports should aim to be concise but not too terse so as to appear incomplete.

A concise radiology report takes effort for those unused to truncating their reports. This has long been recognised; nearly 400 years ago, the polymath Blaise Pascal wrote, “If I had more time, I would have written a shorter letter”. Reports are written under great and increasing time pressure. Fortunately, conciseness can be learned and, with practice, becomes habitual. It isn’t hard to spot the concise but complete reports of an experienced radiologist.

Hartnung et al [24] highlighted the features of a concise radiology report:

i) Removal of redundancy: With practice, information can be expressed in fewer words by rephrasing and systematically removing words that add nothing to the report.
ii) Omit terms of perception: Phrases like “is noted”, “is present” or “there is” make grammatically complete sentences but add no meaningful information. Such omission creates verb-less ‘sentence fragments’ which radiologists may not like but referrers actually prefer [42].
iii) Relevance: Whilst normal structures should be mentioned for completeness, this list should be terse for brevity. Further conciseness can be achieved by including only negative findings pertinent to the clinical question.

#### Rule 3—Clarity of communication is everything

Clarity is the key to accurate communication of meaning; referrers have long valued this in a radiology report [43]. A clear radiology report has several qualities, listed in Box 2. Vague reports are not necessarily inaccurate but are disliked as they fail in their primary duty. Their inherent lack of meaning adds nothing to patient care, as referrers are unsure as to what action to take. Unnecessarily vague reports are, fundamentally, a disservice to the radiologist themselves, the referrer and, ultimately, to the patient [28, 44].

Of course, it is relatively easy to construct a radiology report when the study is either completely or obviously and specifically abnormal. The problem is that the vast majority (if not all) studies contain a degree of uncertainty as radiological reasoning is probabilistic [45]. Articulating uncertainty in radiology reports without hedging is one of the most difficult aspects of creating an actionable report [46, 47].

Hedging is different from an appropriate expression of uncertainty in that it is “an evasive statement to avoid the risk of commitment” [39], typically using qualifiers, vague modifiers and other hedge vocabulary. Hedging should be specifically avoided when there is a strong degree of certainty [48]. Radiologists may employ hedging defensively, but this doesn’t work either—there is evidence that it doesn’t protect from liability [49].

There are four main ways to express uncertainty constructively. Three are suggested by Audi et al [46]: (1) Use of a standardised lexicon, (2) Use of a Reporting and Data Systems (RADS) grading system where possible and (3) Discuss uncertainty at multidisciplinary team (MDT) meetings. The problem is that standardised lexicons are not in widespread use, grading systems have limited application, and only so many cases can be discussed in a meeting. My personal preference is the fourth approach taken by Hartnung et al [24]. Uncertainty is dealt with in the conclusion using free text to articulate the doubt or “phrasing similar to speaking directly to a provider or presenting at a multidisciplinary conference”. This may include honest personal touches and an invitation to discuss the findings.

#### Rule 4—An unreadable report is dangerous

Radiology reports can be difficult to read, often due to excessive length. Sometimes it is the lack of clarity, sometimes it is the complexity of grammar. Even concise reports can be rendered difficult to read through the use of obscure words, impenetrable jargon and complex sentence construction.

Formatting is important too. Dense blocks of text are uninviting to read, whereas a structured report with subheadings is much clearer. The ACR recommends structured reporting for this reason—information can be easier to locate [1]. This said, structured reports can be problematic; they can be overlong (and hence unreadable), promote commission errors (retained erroneous statements from the template) [16], and they can artificially fragment related-processes, disrupting a cohesive synthesis [24].

The notion of unreadable reports being dangerous is not just hyperbole; they are a potential risk to patient safety. When a referrer skips to the conclusion, they miss huge swathes of information in the body of the report. The conclusion may omit key findings that are of critical relevance for the referrer. Hence, the whole report should be readable; otherwise, key information will be missed. Readable reports means better informed referrers and safer patients.

In addition, patients are increasingly being granted access to their own radiology reports [16]. Whilst the report is primarily for the referrer, an easy-to-understand and readable report is appreciated by patients, who may contact their referrer less if they can access results and understand them [50].

#### Rule 5—Guidance is a minefield but is vital

Providing guidance is a key part of an actionable report. Radiologists frequently recommend additional tests, up to 30% of the time [1]. But getting recommendations right is one of the most difficult aspects of actionable reporting [24]. Ferris Hall encapsulates the problems succinctly:

“Radiologists make too many recommendations, particularly in patients about whom we have little clinical history. These recommendations are often not helpful, are sometimes inappropriate and occasionally simply wrong. When the recommendation is obvious, it may be resented… Conversely, insecure clinicians may feel medicolegal pressure to act on our suggestions for additional imaging” [39].

Guidance is relatively easy when you know the referrer, you work in the same hospital, have the same specialist interest and attend weekly meetings within them. But even then, it isn’t easy. A recent single-institute survey of 18 PETCT referrers found varying opinions, but some conclusions could be drawn. Advice was most welcome on further radiological tests, sites for biopsy and topics outside the referrer’s area of expertise. Advice was least welcome on specific management advice (especially without explanation) and when phrased in a mandatory fashion [51].

Hartnung and colleagues [24] urge caution in direct recommendations, reserving these for clear-cut and emergency findings. In all other cases, guidance should be softened, using phrases like ‘suggest’ or ‘may consider’ to allow the referrer room for flexibility. Furthermore, guidance should be tailored to the referrer. Generally, the more specialised the referrer, the less they are appreciated. General practitioners (GPs) tend to appreciate specific guidance [22].

This dilemma can be phrased as “The Goldilocks Report”, from the children’s story of Goldilocks and the Three Bears. Famously, the porridge is either too hot, too cold or just right. And the same is true with guidance in a radiology report. The goal is to give neither too much nor too little guidance, just the right amount. Giving guidance to referrers can be improved if radiologists actively seek feedback, listen and learn.

A note of caution. Recommendations should always advance patient care, preferably by being evidence-based or following agreed guidelines [1]. Instructions to perform follow-up imaging, biopsies or even discussion in an MDT meeting should not be used instead of radiological decision-making in the face of uncertainty. Inexperience, under-confidence or lack of knowledge should not translate into redundant recommendations.

Box 1 The rules of actionable reports

1. Answer the clinical question
2. Conciseness is king
3. Clarity of communication is everything
4. An unreadable report is dangerous
5. Guidance is a minefield but is vital

Box 2 Features of clarity in a radiology report

1. Brevity is a prerequisite; rambling reports are rarely clear
2. Use plain language with minimal radiological jargon, particularly in the conclusion
3. Accurate and explicit observations with an absence of ambiguity or vagueness
4. Uncertainty is clearly articulated with a lack of unnecessary hedging
5. Conclusion should be meaningful and actionable

### Step 4—Alert conveyed to referrer

Whilst all reports should be actionable, certain reports need to be communicated more urgently than others, over and above the written report [31]. This is one of the least controversial steps of actionable reporting but ironically one of the least consistently done.

A poll of 96 delegates at the European Congress of Radiology 2026 found widespread variations in practice [52]. Whilst nearly half (49%) had a system of communicating urgent results, 45% of delegates had no formal policy of alerting referrers. Most (89%) delegates would communicate unexpected abnormalities, but only 60% would communicate expected but serious abnormalities. Just 53% would communicate unexpected cancers. Most (53%) don’t categorise their type of alerts at all, and the majority (85%) alert by either telephone or email.

The ACR recommend that IT can reduce the logistical burden of an alerting system, particularly with alerts of medium risk or where immediate action is not required [2]. More urgent reports need an in-person or telephonic conversation, if only to confirm that the report has been seen. On-site radiologists have advantages here; the teleradiologist is at a disadvantage, underlining the need for robust alert systems when radiologists are working remotely.

For non-critical and non-emergency alerts, radiologists need a system of alerts that don’t rely on the radiologist making telephone calls. Many other systems exist; email, secure messaging apps, ordercomms systems, result portals and so forth. An alerting system (1) should be easy and quick for the radiologist, preferably a ‘single click’ IT solution, (2) reliable, (3) similarly easy for the referrer, (4) contain a tracking and auditing loop to ensure results are received.

Institutions have a responsibility to provide a robust urgent alerting system. Ideally, this would be integrated into existing IT systems via an HL7-compatible infrastructure [2, 21, 31]. Not providing an urgent results alerting infrastructure is clearly unacceptable, but, as shown with the ECR poll, it is relatively commonplace.

Radiologists take a double hit without a formal alert system. The time involved reduces productivity but also makes the radiologist liable for ensuring the alert is safely delivered. Busy radiologists are therefore disincentivised to send alerts, depending on staffing levels and attitudes to clinical risk.

### Step 5—Clinical action is taken

A true actionable report has a positive influence on the patient’s further management. As Waite et al [16] highlight, the amount of error in this phase is likely to be vastly underestimated by radiologists, as they rarely get feedback. However, referrers don’t follow radiological recommendations in 20–50% of cases [53–55].

Clinical action or lack of it is known to depend on several factors:

i) Strength of assertion: Referrers are more likely to adhere to report recommendations if they are stated in absolute rather than conditional terms [56]. Radiologists should be mindful that clear action steps should be explicit and not termed in a conditional manner.
ii) Timeliness: If the report arrives after clinical decisions have been made, the time to make an impact has been lost. Results of tests on inpatients sometimes arrive after the patient has been discharged [57].
iii) Correct referrer alerted: The referrer is ultimately liable for taking clinical action, but what if the individual is temporary (e.g., locum, rotating or shift work)? Or if the responsible/named physician is wrong on PACS/RIS initially? If delivered to an incorrect referrer, it should be easy to re-route.
iv) Referrer acts accordingly: Ideally, the referrer agrees with the information in the report and they act accordingly. However, the referrer may be unsure of what to do next, particularly when the recommendations are outside of their field of expertise [58]. Occasionally, the referrer will disagree with the radiologist, either because they feel a different action is necessary based on their opinion or because further clinical information has come to light, which changes the interpretation of the findings.

## Discussion

Actionable reporting is an enlightened approach that can have great benefits to patient care. Construction of an actionable radiology report is crucial, but it is a multistep process. The chances of a radiology report having a positive impact on patient care are vastly improved when each step of the process is enacted with as much care and precision as the construction of the report itself. Vice versa, a perfectly constructed report can have a greatly reduced impact if all the steps are not followed.

Many radiologists teach reporting skills to their residents, largely informally and in the workplace. Formal teaching of reporting skills outside the workplace is, in my experience, much less common. I advocate the principles of actionable reporting (summarised in Fig. 1 and Boxes 1 and 2) as an overarching framework for the reporting skills that are necessary for independent and successful practice. I also recommend these principles to radiological educators looking for guidance in planning their training programmes.

For all the benefits of actionable reporting, the multiple individual steps can be difficult. Healthcare systems are all resource-constrained, and investing in systems that improve workflow can be difficult to fund. Some of the steps take great skill, knowledge and experience on behalf of the individual radiologist. And several of the steps represent quite a deviation from current practice. Overcoming inertia through change management is not always the easiest.

Nevertheless, the more that radiologists embrace actionable reporting, the greater our positive impact on patient care will be. This is not only by providing actionable reports for our referrers but also by having reporting workflows that are much safer, more reliable and more productive.

## Abbreviations

AoMRC: Academy of Medical Royal Colleges
ACR: American College of Radiology
ESR: European Society of Radiology
IT: Information technology
MDT: Multidisciplinary team
RCR: Royal College of Radiologists
